# Activation of Methanogenesis by Cadmium in the Marine Archaeon *Methanosarcina acetivorans*

**DOI:** 10.1371/journal.pone.0048779

**Published:** 2012-11-12

**Authors:** Elizabeth Lira-Silva, M. Geovanni Santiago-Martínez, Viridiana Hernández-Juárez, Rodolfo García-Contreras, Rafael Moreno-Sánchez, Ricardo Jasso-Chávez

**Affiliations:** Departamento de Bioquímica, Instituto Nacional de Cardiología, Tlalpan, México D.F., México; Shantou University Medical College, China

## Abstract

*Methanosarcina acetivorans* was cultured in the presence of CdCl_2_ to determine the metal effect on cell growth and biogas production. With methanol as substrate, cell growth and methane synthesis were not altered by cadmium, whereas with acetate, cadmium slightly increased both, growth and methane rate synthesis. In cultures metabolically active, incubations for short-term (minutes) with 10 µM total cadmium increased the methanogenesis rate by 6 and 9 folds in methanol- and acetate-grown cells, respectively. Cobalt and zinc but not copper or iron also activated the methane production rate. Methanogenic carbonic anhydrase and acetate kinase were directly activated by cadmium. Indeed, cells cultured in 100 µM total cadmium removed 41–69% of the heavy metal from the culture and accumulated 231–539 nmol Cd/mg cell protein. This is the first report showing that (i) Cd^2+^ has an activating effect on methanogenesis, a biotechnological relevant process in the bio-fuels field; and (ii) a methanogenic archaea is able to remove a heavy metal from aquatic environments.

## Introduction

Methanogenesis is the pathway by which ion (H^+^, Na^+^) gradients across the plasma membrane are generated to drive ATP synthesis, with the concomitant production of methane as an end product. Methanogens are strict anaerobes belonging to the *Archaea* domain, which can be found in a broad variety of environments such as anaerobic digesters of sewage treatment plants, landfills, rice paddies, lakes and in the sea sediments, among others [1]. Indeed, these organisms have an essential role in the global carbon cycle by transforming small carbon molecules such as methanol, methylamines, CO_2_+H_2_, formate, CO and acetate into methane. Because heavy metal pollution may develop in some of these habitats, methanogens may be exposed to this environmental stress with the consequent perturbation of the local ecology. Heavy metal pollution of water resources is now a widespread environmental and public health problem, as a result of their elevated toxicity, which may be exacerbated by their potential bio-magnification effect and accumulation throughout the ecological food webs.

Pollution of coastal zones by heavy metals such as Cd, Pb, Hg and Ni, is a major environmental problem in some regions of the world [2]. Once in the marine environment, these contaminants accumulate in sediments [3]. Cadmium ocean pollution and mobilization has increased exponentially up to 300 thousands per decade, where 40% of the total current pollution derives from anthropogenic activities [4]. In some coastal zones in the Gulf of Mexico, up to 2,550 µg L^−1^ (22.6 µM) of cadmium has been found, a value far higher than permissible [5]. In other seas and oceans around the world, cadmium concentrations up to 20.5 µg L^−1^ and18, 400 µg g^−1^ in waters and sediments, respectively, have been determined [4]. These cadmium pollution values highlight the importance of determining the toxicity of cadmium in organisms found in sediments, methanogens among them. Depending on the physicochemical environmental characteristics and microbial metabolism, heavy metal contaminants may be released from sediments back into the water. Under such situations, marine sediments may become a secondary source of pollution.

Most studies of microbial communities in the ocean have focused on bacterial diversity in marine sediments and the long-term impact brought about by heavy metals exposure [6]. There are few works about the toxic effect of heavy metals on methanogens in sludge [7] and laboratory strains [8], [9]. Remarkably, it has been described that low concentrations of heavy metals are not toxic for methanogens in the sludge, but on the contrary they induce increased methane production [10]. This finding has not been further explored and hence the living components of the consortia affected by the metal have not been identified, and the biochemical mechanisms involved have not been elucidated. In the present work, the marine archaeon *Methanosarcina acetivorans* was used as a model to assess the effect of cadmium on methanogenesis.

## Materials and Methods

### Chemicals

Acetate kinase from *M. thermophila*, deoxyribonuclease I from bovine pancreas (DNAse I), acetyl-CoA, coenzyme A, acetyl phosphate, ATP and NADH were purchased from Sigma Chem. (St. Louis, Mo, USA). Phosphoenolpyruvate, pyruvate kinase and NAD^+^-lactate dehydrogenase (both enzymes from rabbit muscle) were from ROCHE (Germany). Absolute methanol, acetate, and CdCl_2_ were of analytical grade.

### Growth conditions

*Methanosarcina acetivorans* C2A strain, kindly provided by Prof. James G. Ferry (Pennsylvania State University, USA), was cultured under anoxic conditions in the regular high salt medium (HS-medium) described elsewhere [11]. Briefly, Milli Q water was placed into an anaerobic chamber (COY laboratory products, Grass Lake, Michigan, USA) filled up with 80% N_2_, 15% CO_2_ and 5% H_2_. Then, the following salts were added, in g/L: NaCl, 25.4; NaHCO_3_, 3.8; KCl, 1.0; MgCl_2_, 11; CaCl_2_, 0.2; NH_4_Cl; 1.0 and KH_2_PO_4_, 0.27 and resazurin, 0.001% (w/v) as redox indicator. Vitamin and trace mineral solutions were both added at 1% (v/v) as reported by Sowers et al [12]. Medium was bubbled with the mix of gases describe above for 2–3 h. Next, 120 mM methanol or 100 mM acetate were added as carbon source, followed by 1 g cysteine-HCl (8.2 mM) and 0.25 g Na_2_S•9H_2_O (1.04 mM) to ensure complete chemical reduction of growth media. Final pH was 6.8–7.0. 50 mL of medium were poured into 100 mL serum-like bottles (Virmel, Mexico), sealed with a butyl rubber stopper (Virmel, Mexico) and secured with an aluminum crimp collar (Virmel, Mexico). Medium was autoclaved at 121°C for 30 min. After autoclaving a precipitate was formed in the culture media but disappeared after 24 h, approximately. Cultures were started by adding fresh cell inocula and further incubating at 37°C without shaking. Growth was determined by measuring changes in absorbance at 600 nm.

### Metabolites content determination

The concentration of the reduced cysteine and sulfide in the fresh medium was determined post column with DTNB (5, 5′-dithiobis-(2-nitrobenzoic acid) by HPLC as described elsewhere [13]. Briefly, 1 mL of fresh medium was taken with a syringe from the anaerobic culture bottles and immediately filtered through a 0.45 µm (pore diameter) filter unit (Millex-HV, Millipore, Ireland) and injected (50 µL) into the HPLC apparatus. The concentration of thiol-groups was calculated by using the DTNB molar extinction coefficient of 13.6 mM^−1^ cm^−1^. Sulfide was also determined spectrophotometrically by the methylene blue formation as described by King and Morris [14] with some modifications: in 10 mL anaerobic bottles sealed with a butyl rubber stopper and secured with an aluminum crimp collar, 23.7 mM zinc acetate, 60 mM NaOH, 0.18 mM N,N-dimethyl-*p*-phenylenediamine (DMPD) dissolved in 5 N HCl and 0.1 mL of culture medium, or different amounts of sulfide, were added by using a syringe and mixed until homogeneity. Then, 2.8 mM FeCl_3_ was added and incubated at room temperature for 30 min for color development (methylene blue formation). Final volume was 2.5 mL. Samples were measured at 670 nm under anoxic conditions in an anaerobic chamber. The sulfide content-absorbance relationship was linear up to 350 nmol.

Methane production and methanol were determined by gas chromatography (Shimadzu GC2010 apparatus), equipped with a capillary column HP-PLOT/U of 30 m length, 0.32 mm I.D. and 10 µm film (Agilent, USA) and flame ionization detector. Methane standard curve was carried out as reported by Sowers [12]. For determination of extracellular acetate, aliquots withdrawn from cell cultures with acetate were centrifuged. Samples of the cell-free supernatant were diluted with a buffer containing 50 mM Hepes (4-(2-hydroxyethyl)-1-piperazineethanesulfonic acid), 10 mM MgCl_2_ and 1 mM EGTA (ethylene glycol tetraacetic acid) at pH 7.5. The mixture reaction assay also contained 5 mM ATP, 2 mM phosphoenolpyruvate, 0.25 mM NADH, and acetate kinase, pyruvate kinase and lactate dehydrogenase, which were added in excess (>5 U, each) to ensure the complete phosphorylation of acetate coupled to NADH oxidation.

### 2.4 Cadmium exposure

To determine the effect of cadmium on growth, acetate and methanol cultures were carried out in the presence of different total CdCl_2_ concentrations (0, 1, 10, 25, 50 and 100 µM) and the optical density was determined at 600 nm. Such range of cadmium concentrations has been reported to be toxic for a broad range of microorganisms from fresh [15] and marine waters [16].

To determine the effect of cadmium on methane biosynthesis, metabolically active cell cultures in the early stationary growth phase with acetate (at the 10–12 day of culture, with 8 mM of remaining acetate) or methanol (at the 3–4 day of culture, with 5 mM remaining methanol), respectively, were initially subjected to depletion of methane formed by gassing the culture bottles with sterile N_2_. Thereafter, 1, 10 or 100 µM total CdCl_2_ or other heavy metals (Zn^2+^, Cu^2+^, Hg^2+^, Fe^2+^ and Co^2+^) were added to the cultures and methane production was determined from the head space at different short times up to 12 min. For longer periods of methane synthesis (up to 60 min) in the presence of Cd^2+^, 20 mM acetate was further supplemented to the incubation medium.

### 2.5 Enzyme activity assays

Cell cultures of 750 mL grown on acetate were harvested under anoxic conditions in the early stationary phase by centrifuging at 3,000× g for 10 min and washed once with 4 volumes of a solution containing 50 mM Tris-HCl pH 7.5, 20 mM MgCl_2_ and 0.02 mM ZnCl_2_. Then, the cell pellet was re-suspended in lysis buffer (0.1 M Na-phosphate, pH 8.0 *plus* some grains of DNAse I), stirred strongly for 5 min and centrifuged at 70,000× g for 30 min. The supernatant (cytosolic fraction; yield 50–100 mg protein) was kept on ice and used immediately for enzyme activities determination. All activities (except for carbonic anhydrase) were determined in the direction of acetate degradation in 50 mM Na_2_-Hepes and 10 mM MgCl_2_ buffer at pH 7.0 and 27±2°C, in the presence of different CdCl_2_ concentrations. In all cases, the reaction assay was started by adding the enzyme (*i.e*. the cytosol-enriched fraction).

Acetate kinase (AK) activity was determined in cytosolic enriched-fractions of 50–75 µg protein in a reaction medium that also contained 5 mM ATP, 20 mM acetate, 0.2 mM NADH, 2 mM phosphoenol pyruvate and 10 U of both, pyruvate kinase and lactate dehydrogenase. One unit of enzyme (U) is the amount of active enzyme required to transform/produce 1 µmol of substrate/product in 1 min. Phosphotransacetylase (Pta) activity was determined as follows: 3–5 µg of cytosolic protein were incubated in the Hepes-Mg buffer with 5 mM acetyl-phosphate and 160 µM CoA; aliquots were withdrawn at different times (from 5 up to 60 s), mixed with 0.1 M phosphate buffer and 1 mM DTNB and the reaction monitored at 412 nm (representative traces are shown in figure S1).

CODH/acetylCoA synthase activity (CODH/AcCoAs) was determined anaerobically by mixing 10–25 µg protein with 80 µM acetyl-CoA and measuring the release of CoA with DTNB at 412 nm. As several different enzymes may release CoA from acetyl-CoA, this activity was also specifically determined by measuring the CO-dependent reduction of methyl viologen as reported elsewhere [12]. Briefly, in an anaerobic sealed bottle the Hepes-Mg buffer +0.5 mM methyl viologen was saturated with CO by bubbling the gas for 30 min (reaction mixture); then, 1.2 mL of reaction mixture was poured into a sealed glass cuvette previously purged with CO. The reaction was started by adding 50 µg of protein and followed at 603 nm. As control of the CODH/AcCoAs activity, the cytosolic fraction was gently mixed with air for 10 min, with the remaining activity being lower than 53% (n = 2) of that determined with saturating CO or acetyl-CoA (representative trace is shown in figure S2). Also, 0.5 mM sodium cyanide inhibited the reduction of methyl viologen coupled to CO oxidation by 85±8% (n = 3) as reported previously for the enzyme from *M. thermophila* [17].

Carbonic anhydrase (CA) activity was determined by incubating 2.5–5 µg of cytosolic protein with 100 mM Na-bicarbonate in a sealed 10 mL bottle with rubber stopper. To detect the CO_2_ formation, 5 µL of the head space was taken and injected at different times (0, 30, 60 and 120 s) in a gas chromatograph equipped with a Thermal Conductivity Detector. CO_2_ formed in assay reaction buffer without enzyme and with boiled enzyme was subtracted from the CO_2_ formed with enzyme (representative traces are shown in figures S3 and S4).

### 2.6 Cadmium removal and accumulation

Cells were harvested and washed as indicated above with 200 volumes of a solution containing 50 mM Tris-HCl, 2 mM MgCl_2_ and 2 mM EGTA (TME buffer) at pH 7.5; the pellet was re-suspended in fresh buffer to give 5–10 mg protein/mL and frozen at −70°C until use. Aliquots of the cell suspension were digested with H_2_SO_4_+HNO_3_ (1∶3) for 2 h at 100°C and the intracellular cadmium content determined by atomic absorption spectrophotometry (Varian Spectra AA 640).

### 2.7 Ultrastructure analysis

Methanol-grown cells with or without 100 µM CdCl_2_ were fixed by immersion in glutaraldehyde (3%, v/v, in phosphate buffer, pH 7.4), after removal from the culture medium, and dehydrated in graded ethanol. Samples of 1 mm^2^ containing the cells were cut out in cross section with a diamond knife and embedded in 1∶1 epoxy resin. To determine cadmium and sulfur localization inside the cells, atomic-resolution high angle annular dark-field scanning-transmission electron microscopy (HAADF-STEM) was used as reported previously [18].

The protein content was determined after cells were washed once with TME buffer by the Biuret method with bovine serum albumin as standard as described previously [13]. For the statistical analysis of the data, the Student's t-test or a two way ANOVA and Bonferroni post analyses were performed using the Graph Pad PRISM version 5.01 software.

## Results and Discussion

### Cadmium solubility and effect on cell growth

Because cysteine and sulfide present in the culture medium bind the cadmium added with high affinity, the soluble free Cd^2+^ concentrations were estimated (see Table I) by using the program Chelator [19] and the following physico-chemical conditions. The concentration of the reduced cysteine and sulfide in the medium determined experimentally were for cysteine 1.7±0.03 mM and for sulfide 1.21±0.4 and 0.95±0.03 mM as determined by HPLC (DTNB) and spectrophotometrically (methylene blue), respectively (mean ± SE, n = 4); the ionic strength = 0.77, pH = 7.0 and temperature = 36°C. The *log* values of the equilibrium constants (*K_eq_*) for the association of the complexes were 13.4 and 20.13 for Cys-cadmium and Cys-Cd-Cys, and 6.1 for sulfide-cadmium [20].

**Table 1:** Methane production and cadmium accumulation in *M. acetivorans* cultured on acetate or methanol.

Total [CdCl_2_] µM	Estimated Free [Cd^2+^] pM	mg of total protein/culture		Methane produced mmol/240 h (acetate) or 96 h (methanol)		Cd removed and accumulated nmol/total cell protein		% of cadmium removed 240 h(acetate) or 96 h (methanol)	
		acetate	methanol	acetate	methanol	Acetate	methanol	acetate	Methanol
0	0	5.2±1.04	10.2±3	4.5±0.3	4.1±0.13	0±0	0±0	0±0	0±0
1	2.1	5.8±0.4	9.6±3.2	4.4±0.5	4.08±0.03	8.3±4.7	10.8±6	16±4^c^	22±13
10	21.4	5.6±0.1	8.9±1.9	4.5±0.27	4.1±0.03	40±10	99±20	8±2^c^	30±23
25	54	5.1±1.5	7.9±1.5	4.6±0.2	4.3±0.1	475±109	459±220	38±9	36±18
50	109	5.9±0.6	9.6±3.8	4.6±0.2	4.1±0.03	1387±225	940±326^b^	55±9	38±23
100	226	6.4±0.1	8.9±2.5	4.7±0.3	4.1±0.02	3453±1430^a^	2054±929^b^	69±35	41±20

Data shown were obtained from cell cultures at the end of the growth curve. Values are the mean ± SD of at least 4 cultures from different batches.

a : P<0.05 *vs* acetate-grown cells at any other concentration of cadmium;

b : P<0.05 *vs* methanol-grown cells at any other concentration of cadmium;

c acetate-grown cells *vs* 25, 50 and 100 µM cadmium, using the Student's t-test.

**Figure 1:**
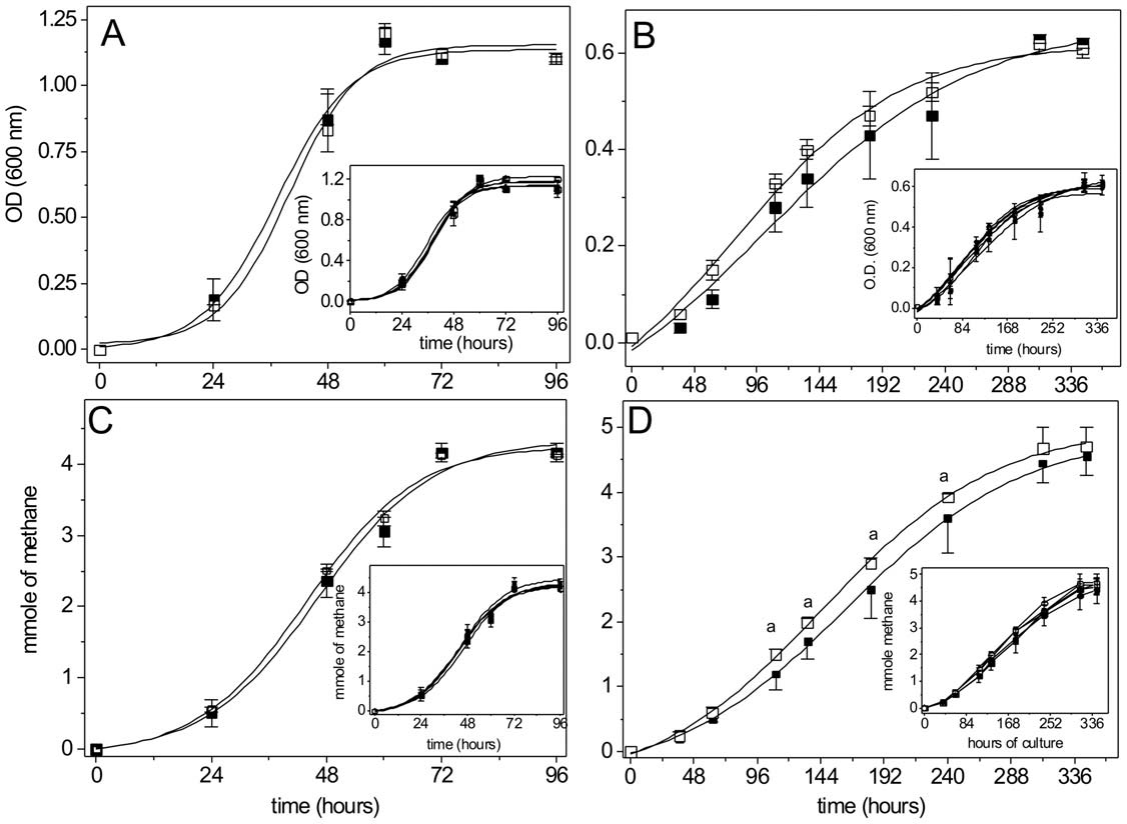
Growth curves and methane synthesis of *M. acetivorans* cultured on methanol (A, C) or acetate (B, D), respectively, and in the absence (filled squares) or presence of 100 µM CdCl_2_ (open squares). Values represent the mean ± SD of at least 4 different cell batches. ^a^: P<0.05 *vs* control curve without cadmium using two way ANOVA. Inset; curves with 1 (filled circles), 10 (filled triangles), 25 (open squares) and 50 (open circles) µM CdCl_2_.

Cells cultured on methanol showed similar growth either in the absence or presence of up to 100 µM total CdCl_2_ (Fig. 1A and inset). With acetate, growth was slightly faster in cultures with 100 µM cadmium during the exponential phase (Fig. 1B and inset). To undoubtedly establish that the turbidity increase induced by cadmium was indeed reporting cell growth in acetate cultures, the growth rate (GR) and the duplication time (DT) were determined by using the curve of methane production *vs* time and assuming that methane production is proportional to the number of living cells present in the culture. The duplication times were similar to those reported previously for *M. acetivorans* [11]. No significant differences in GR values (0.064±0.003 *versus* 0.0625±0.003 h^−1^) and DT values (10±2.3 h *versus* 11±2.7 h) were found for cells cultured in methanol without or with 100 µM total Cd^2+^. In contrast, in acetate cultures the GR value was significantly higher in cultures with 100 µM Cd^2+^ (0.028±0.004 *versus* 0.030±0.006 h^−1^; n = 5, P<0.05). DT did not significantly changed (26±3 *versus* 24±2 h; n = 5) for cells cultured without or with 100 µM Cd, respectively (Fig. 1). Furthermore, two way ANOVA analyses on the global data showed that cadmium exerted a positive effect on the growth curve with acetate but not with methanol as carbon source (Figs. 1C and 1D).

The rate of methane production in acetate cultures with 100 µM total CdCl_2_ was slightly but significantly higher than in its absence in the time-period from 110 up to 230 h of culture (Fig. 1D). It is worth noting that the methane yield, at the end of the growth curves (96 and 244 h for methanol and acetate, respectively), was the same under all conditions, because the total amount of carbon source added was identical (Table 1): 4.1±0.13 (control) and 4.1±0.02 mmol methane (+100 µM CdCl_2_) for methanol and 4.5±0.3 (control) and 4.7±0.3 mmol methane (+100 µM CdCl_2_) for acetate. On the other hand, when 500 µM total CdCl_2_ was added to cultures with acetate or methanol as carbon source, no growth or methane synthesis were detected (data not shown) indicating that these high cadmium levels were indeed extremely toxic to the cells.

### Effect of cadmium on methane synthesis

**Figure 2:**
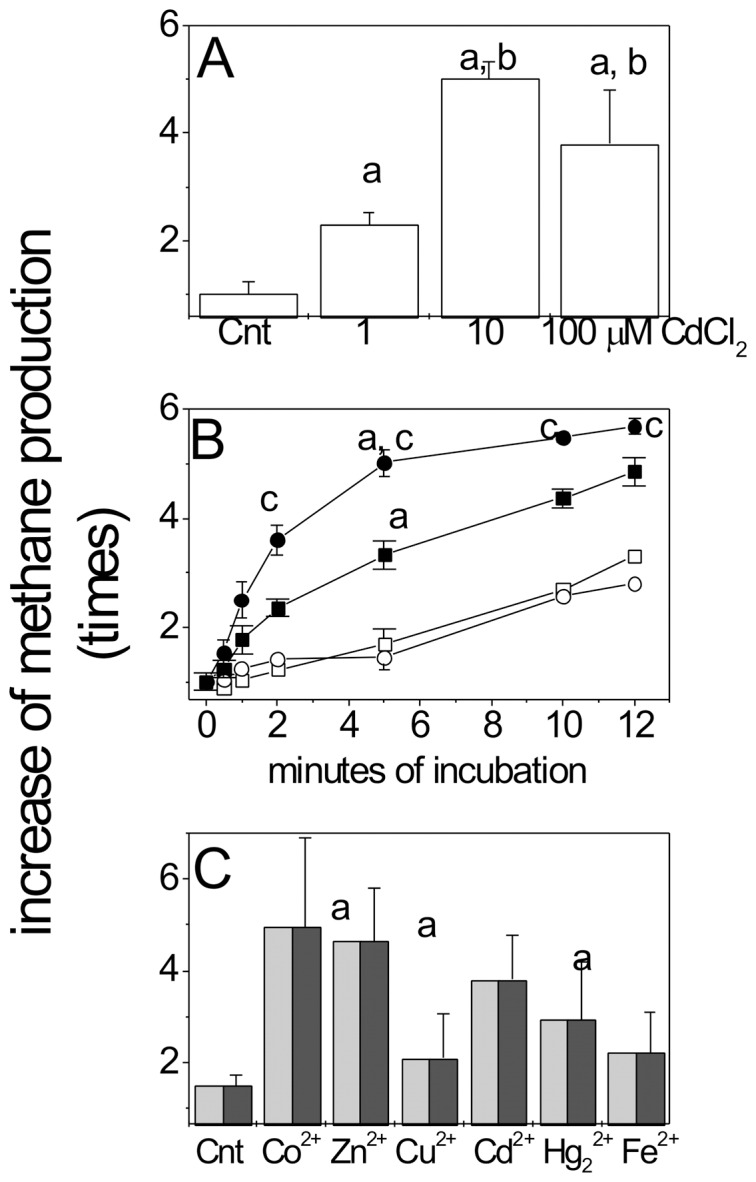
Activation of methane synthesis by cadmium. (A) 1, 10 and 100 µM of CdCl_2_ were added and methane production was determined after 5 min in acetate-grown control cells. (B) Short-term methane synthesis in the absence (open symbols) or presence (filled symbols) of 10 µM CdCl_2_ in methanol- (▪) and acetate-grown cells (•). These experiments were started after thoroughly purging the flasks and adding the indicated CdCl_2_ concentrations (time-point equal to zero). (C) Activation of methane synthesis by other heavy metals. Acetate-grown cells cultures were incubated for 5 min in the absence or presence of 100 µM of the metals indicated. At t = 0 (before metal addition), the methane remaining in the bottle cultures was 8.8±1.2 µmol methane *per* culture. P<0.05 using the Student's t-test for non-paired samples for ^a^ *vs* control (without cadmium or other metal ion); ^b^ *vs* cells exposed to 1 µM cadmium; ^c^ *vs* methanol cultures exposed to cadmium.

For short-term experiments, cultures of cells in the early stationary growth phase (10 days for acetate- and 4 days for methanol-grown cells) were incubated with CdCl_2_ at 25–27°C. The concentrations of acetate and methanol remaining in the cultures were 8±3 mM (400±150 µmol acetate; n = 5) and 5±1 mM (250±50 µmol methanol; n = 5), respectively. Under these conditions, cadmium exerted a remarkably stimulating effect on the synthesis of methane in control cells not previously exposed to Cd^2+^; the most potent activation was reached at 10 µM total CdCl_2_ (Fig. 2A). Moreover, the rate of the methane production increased 9 and 6.5 fold for acetate- and methanol-grown cells, respectively, in 2 min (Fig. 2B).

After 10 min of incubation the methane produced, in the absence or presence of 10 µM total CdCl_2_, by stationary acetate-grown cells was 18±3 and 26±4 µmol methane, and after 60 min was 24±2 and 43±8 µmol methane, respectively (Fig. S5). Hence, the methane produced was the same regardless the carbon source concentration, sub-saturating or growth-limiting (8 mM acetate, Fig. 2B) for the 10 min experiments and saturating (20 mM acetate, Fig. S5) for the 60 min experiments.

Activation of methanogenesis was not exclusive for cadmium, since also 100 µM of the essential trace metals Co^2+^ or Zn^2+^ had a similar effect, whereas Cu^2+^ and Fe^2+^, also essential trace metals, or Hg^2+^ were poor activators of the methane production (Fig. 2C).

These data suggested that the activation of methane production by cadmium was not due to the precipitation of sulfur that may be toxic for the cell, as copper, iron and mercury can also form complexes with sulfur; in fact, insoluble complexes were apparent with iron. A copper inhibitory effect was previously described for methanogenesis derived from anaerobic sludge digestion [10], [21]. On this regard, a positive effect of pM concentrations cadmium on growth presumably due to activation of CA was reported for *Thalassiosira weissflogii*, a diatom that also comes from the marine habitat [22]. Interestingly, acetate-grown *M. acetivorans* cells have significant higher AK, Pta, CODH/AcCoAs and CA protein content than methanol-grown cells [23], [24]. Hence, the influence of cadmium on enzymes activities from the upper part of the aceticlastic pathway, which have not been previously determined in *M. acetivorans*, was here examined (Table 2).

**Table 2:** Effect of cadmium on enzyme activities of the acetoclastic pathway upper part from *Methanosarcina acetivorans*.

[CdCl_2_] µM	Acetate kinase times	Phosphotranacetylase times	CODH/AcCoA synthase times		Carbonic anhydrase times
			with Acetyl-CoA	with CO	
0	1	1	1	1	1
0.01	1.38±0.27	Not determined	1.0±0.1	1.03±0.11	2.9±0.8 **
0.1	1.35±0.18**	Not determined	1.02±0.3	0.66±0.21	4.9±3.2 *
1	1.1±0.16	0.73±0.15	0.98±0.18	0.60±0.16	4.2±2.7 *
10	Not determined	0.77±0.17	0.74±0.2	0.5±0.16	1.7±0.8
100	ND	0.47±0.08	ND	ND	ND

All activities were determined by using freshly prepared cytosolic fraction as described in the Methods section. Values are the mean ± SD of at least three independent preparations.

Control activities were for AK: 0.75±0.21 U/mg protein (n = 4); for Pta: 1.48±0.8 U/mg protein (n = 4); for CODH/AcCoA synthase with acetyl-CoA: 0.37 U±0.12 U/mg protein (n = 5); and with CO: 0.68±0.11 U/mg protein (n = 3); CA: 26±12 U/mg protein.

* P<0.05 *vs* control for independent samples;

** P<0.05 *vs* control for paired samples. ND: Not determined.

AK activity was 10 fold lower (see legend to Table 2 for values) than that reported for the enzyme from *M. thermophila* [25]; the AK activity slightly increased (25–30%) by 10 µM total cadmium. This cadmium activating effect is intriguing because no metal has been reported to be required for AK activity in *M. thermophila* [26]. Pta activity under our conditions was 15 times lower than that reported for the enzyme from *M. thermophila* [27], whereas the CODH/AcCoAs activity determined in the present work was 10 times higher than that reported for the enzyme from *M. thermophila* [17]. The last two enzymes were not activated by 0.01–10 µM total CdCl_2_, but they were rather partially inhibited (Table 2). With a novel strategy to determine CA activity which was based on measuring by gas chromatography the CO_2_ produced, the *M. acetivorans* CA showed a higher activity than that reported by semi-quantitative electrometric method at alkaline pH for the *M. thermophila* enzyme [28] and marked activation by 1–10 µM total cadmium (Table 2).

*Methanosarcina* CA is promiscuous respect to the metal bound into its active centre, because the presence of zinc, cobalt and even iron has been reported for this enzyme in *M. thermophila* and *M. acetivorans* [29], [30]. Indeed, the recombinant purified CA showed activity even with Cd^2+^ [31]; hence, cadmium might also be able to bind and activate CA *in vivo*. Thus, activation of CA and AK by cadmium may be involved in the higher methane production in acetate-grown cells. Another possible explanation for the stimulation of the methane production was that cadmium uncoupled the methanogenic pathway by collapsing the ion gradient across the plasma membrane. However, the total protein determined at the end of culture in cells grown with cadmium suggested that ATP content was not compromised. On the other hand, cadmium activation of methanogenesis suggested metal internalized into cells; hence, the cadmium removal from cultures by cells was determined.

### Cadmium removal

Under our culture conditions, in which the cysteine and sulfide concentrations were high, the added micromolar CdCl_2_ concentrations yielded free Cd^2+^ concentrations in the pM range (Table 1). It is known that organic and inorganic sulfur may attenuate the toxicity of Cr (VI) in yeasts isolated from industrial wastes [32]. Hence, the low toxicity of cadmium in *M. acetivorans* may be due to the low free Cd^2+^ available in the medium. Nevertheless, cells surprisingly removed up to 70% and 40% of total added cadmium from the medium in the cultures with acetate or methanol, respectively (Table 1). In this regard, with 100 µM added CdCl_2_, an accumulation of 0.54 and 0.23 µmol cadmium/mg cell protein (Table 1) was determined for acetate and methanol-grown cells, respectively, which were harvested after 10 or 4 days culture and washed once with an EGTA (*e.g.*, potent metal ion chelating agent)-containing buffer. The cell-free culture medium contained 1.4±0.1 µM total cadmium. In turn, 0.04±0.01 and 0.1±0.03 µmol total cadmium/mg cell protein were found in the supernatant after the EGTA-washing treatment in acetate- and methanol-grown cells, respectively (i.e., adsorbed Cd^2+^ to the cell outer layers), revealing that most of the cadmium associated with the cells was indeed intra-cellularly trapped.

**Figure 3:**
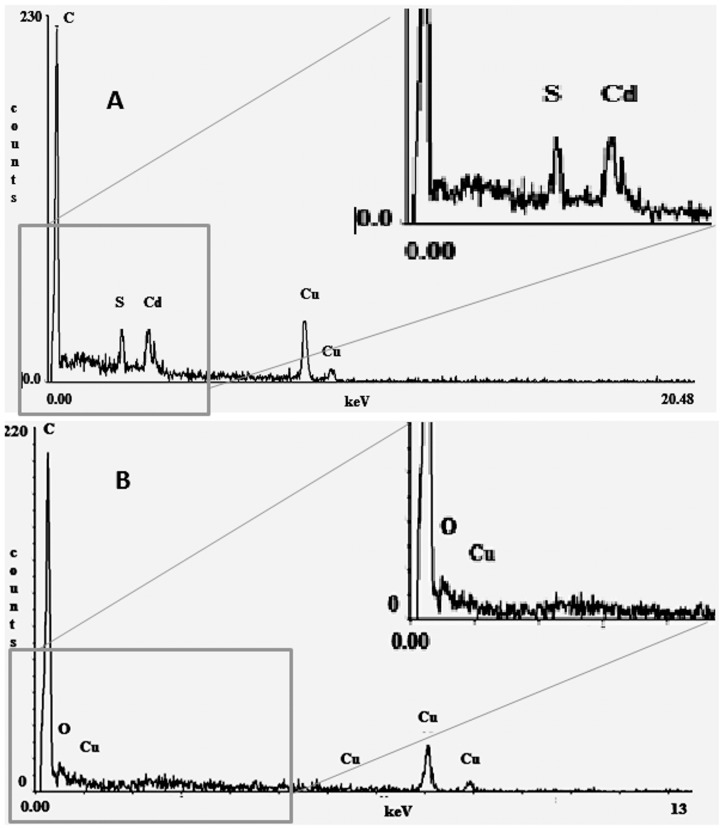
Identification of cadmium clusters in *M. acetivorans*. Spectral analysis by HAADF-STEM from methanol-grown cells with 100 µM CdCl_2_ (A) or without cadmium (B). C: carbon; O: oxygen, Cu: cupper; S: sulfur; Cd: cadmium.

Due to the extremely low free Cd^2+^ concentration, it seems likely that the complexes formed between cadmium and sulfur compounds, and not the free Cd^2+^, were the species that preferentially entered into cells (Table 1). To further demonstrate that cadmium was indeed inside the cells, methanol-grown cells cultured in 100 µM total CdCl_2_ were prepared as previously reported [18] for HAADF-STEM. Although the images were diffused (Fig. S6), high intracellular contents of electro-dense grains of cadmium and sulfur were definitively identified (Fig. 3). A similar accumulation profile has been reported for cobalt, where the complexes cobalt-chloride and cobalt-citrate are the forms that preferentially are retained in the granular sludge and affect methane production [33]. Such high capacity for cadmium removal in *M. acetivorans* suggests that this *Archaea* may have developed strategies to contend against heavy metals different to those reported for the eubacteria domain, which are mainly based on the extrusion of the metal by means of pumps that use ATP as motive force [34].

It has been documented that an excess of zinc, copper, or cobalt, all essential heavy metals, inhibit methanogenesis [9], [31], [35], [36]. In contrast, cadmium seems to be less toxic for methanogenesis in the granular sludge [10], although the free metal concentration or the organic complexes formed were not described. In the present study the free Cd^2+^ concentration in the culture medium was estimated to be in the pM range. This suggested that *M. acetivorans* has very high affinity transporters for Cd^2+^ or that the metal ion may permeate the plasma membrane as a complex with the external cysteine and sulfur.

### Concluding remarks

Despite the very low concentration calculated of free Cd^2+^, this non-essential heavy metal was able to activate a biological process, *i.e.*, methanogenesis in *M. acetivorans*, due in part to a direct activation of acetoclastic pathway enzymes. *M. acetivorans* removed and accumulated cadmium; hence, *M. acetivorans* may become a suitable model for studying the effect of heavy metals on marine methanogens and its mechanisms of heavy metal resistance in the *Archaea* domain. Moreover, further optimization of the enhanced methane production by cadmium, and other heavy metals, may place this process in the biotechnological leading frontier for generation of biogas.

## Supporting Information

Figure S1 **Activity of phosphotransacetylase from** ***M. acetivorans*** **.** An aliquot of the cytosolic fraction was incubated with the substrates acetyl-Pi and CoA in the absence ((▪) or presence of 0.1 (•), 1 (▴) or 10 (▾) µM total CdCl_2_. In the absence of protein the CoA concentration remained constant (□).
(TIF)

Figure S2 **CODH/AcCoAs complex activity from** ***M. acetivorans*** **;** representative traces of the activity by adding cytosolic fraction (containing the enzyme complex, ferredoxin and THMPT) and 80 µM AcCoA. Representative trace with: 125 (□), 62 (x) and 25 (▪) µg of cytosolic fraction without cadmium in the absence or presence of 0.01 (•), 0.1 (▴), 1 (▾) and 10 µM total CdCl2 (⧫).
(TIF)

Figure S3 **Formation of CO_2_ by CA from** ***M. acetivorans*** in the absence (chromatogram A), or presence of 1 µM CdCl_2_. In chromatogram C, cytosolic fraction was previously heated. The different peaks shown represent consecutive sample injections at different times. It should be noted that *y*-scale is higher for chromatogram B. Under these conditions, CA activity was linear for at least 1 min (Fig. S4). Reverse reaction in turn, produced less reliable values by this method (not shown).
(TIF)

Figure S4 **Activity of CA in the cytosolicfraction of** ***M. acetivorans*** in the absence (▪) or presence of 0.01 (•), 0.1 (▴), 1 (▾) or 10 µM total CdCl_2_ (⧫). A representative data with heated cytosolic fraction in presence of 0.1 µM CdCl_2_ is also shown (□).
(TIF)

Figure S5 **Activation of methanogenesis by cadmium.** Cultures on acetate were purged by passing N2 for 5 min. Then, samples of the head space were withdrawn from the cultures at 0 and 5, 10, 20, 30 and 60 min of incubation with 0 (filled symbols) or 10 µM CdCl_2_ (open symbols) for GC analysis. These experiments were started with the addition of 20 mM acetate. Values are the mean ± SD of 3 independent cell preparations. P<0.05 using the Student's t-test for ^a^ *vs* control (without cadmium).
(TIF)

Figure S6 **Intracellular cadmium clusters in** ***M. acetivorans*** **.** HAADF-STEM projection images of methanol-grown cells cultured in methanol in the absence (A) or in the presence of 100 µM CdCl_2_ for 5 days (B). The image in B revealed cadmium grains along the cell (white spots).
(TIF)

Text S1 **Methods and Results.**
(DOCX)

## References

[1] Ferry JG, Kastead KA (2007) Methanogenesis, In: Archaea, Molecular and cellular biology Ed. Ricardo Cavicchioli (ASM press), Washington, DC. USA. pp 100–123.

[2] Cesar A, Marín A, Marin-Guirao L, Vita R, Lloret J, et al (2009) Integrative ecotoxicological assessment of sediment in Portmán Bay (southeast Spain). Ecotoxicol Environ Saf 72: 1832–1841. 19615746 10.1016/j.ecoenv.2008.12.001

[3] Gillan DC, Danis B, Pernet P, Joly G, Dubois P (2005) Structure of sediment-associated microbial communities along a heavy-metal contamination gradient in the marine environment. Appl Environ Microbiol 71: 679–690. 15691917 10.1128/AEM.71.2.679-690.2005 PMC546797

[4] Simpson WR (1981) A critical review of cadmium on marine environment. Progr Oceanogr 10: 1–70.

[5] Villanueva S, Alfonso V (1992) Heavy metals in the coastal zone from Gulf of Mexico and Mexican Caribbean: a review (Metales pesados en la zona costera del Golfo de México y Caribe Mexicano: una revisión). Red Rev Cient Amér Latin Carib Esp y Port 8: 47–61 (Web page: http://redalyc.uaemex.mx/src/inicio/HomRevRed.jsp?iCveEntRev=370. Accessed 2012 Oct 5).

[6] Toes AC, Finke N, Kuenen JG, Muyzer G (2008) Effects of deposition of heavy-metal-polluted harbor mud on microbial diversity and metal resistance in sandy marine sediments. Arch Environ Contam Toxicol 55: 372–385. 18273665 10.1007/s00244-008-9135-4

[7] Florencio L, Field JA, Lettinga G (1994) Importance of cobalt for individual trophic groups in an anaerobic methanol-degrading consortium. Appl Environ Microbiol 60: 227–234. 8117078 10.1128/aem.60.1.227-234.1994 PMC201293

[8] Jarrell KF, Saulnier M, Ley A (1987) Inhibition of methanogenesis in pure cultures by ammonia, fatty acids, and heavy metals, and protection against heavy metal toxicity by sewage sludge. Can J Microbiol 33: 551–554. 3621086 10.1139/m87-093

[9] Mori K, Hatsu M, Kimura R, Takamizawa K (2000) Effect of heavy metals on the growth of a methanogen in pure culture and co culture with a sulfate-reducing bacterium. J Biosci Bioeng 90: 260–265. 16232854 10.1016/s1389-1723(00)80079-1

[10] Altaş L (2009) Inhibitory effect of heavy metals on methane-producing anaerobic granular sludge. J Hazard Mater 162: 1551–1556. 18640779 10.1016/j.jhazmat.2008.06.048

[11] Sowers KR, Baron SF, Ferry JG (1984) *Methanosarcina acetivorans* sp. nov., an acetotrophic methane-producing bacterium isolated from marine sediments. Appl Environ Microbiol 47: 971–978. 16346552 10.1128/aem.47.5.971-978.1984 PMC240030

[12] Sowers KR (1995) Methanogens: growth and identification. *In:* *Archaea*- A Laboratory Manual Robb FT, Sowers KR, DasSharma S, Place AR, Schreier HJ, Fleischmann EM (eds.) Cold Spring Harbor Press, Cold Spring Harbor, NY. pp 200–245

[13] Lira-Silva E, Ramírez-Lima IS, Olín-Sandoval V, García-García JD, García-Contreras, et al (2011) Removal, accumulation and resistance to chromium in heterotrophic *Euglena gracilis* . J Hazard Mater 193: 216–224. 21831522 10.1016/j.jhazmat.2011.07.056

[14] King TE, Morris RO (1967) Determination of Acid-Labile Sulfide and Sulfhydryl Groups. Methods Enzymol 10: 634–641.

[15] Madoni P, Romeo MG (2006) Acute toxicity of heavy metals towards freshwater ciliated protists. Environ Pollut 141: 1–7. 16198032 10.1016/j.envpol.2005.08.025

[16] Wang M, Wang WX (2011) Cadmium sensitivity, uptake, subcellular distribution and thiol induction in a marine diatom: Recovery from cadmium exposure. Aquat Toxicol 101: 387–395. 21216349 10.1016/j.aquatox.2010.11.012

[17] Abbanat DR, Ferry JG (1990) Synthesis of acetyl coenzyme A by carbon monoxide dehydrogenase complex from acetate-grown *Methanosarcina thermophila* . J Bacteriol 172: 7145–7150. 2123865 10.1128/jb.172.12.7145-7150.1990 PMC210839

[18] Mendoza-Cózatl DG, Rodríguez-Zavala JS, Rodríguez-Enríquez S, Mendoza-Hernández G, Briones-Gallardo R, et al (2006) Phytochelatin-cadmium-sulfide high-molecular-mass complexes of *Euglena gracilis* . FEBS J 273: 5703–5713. 17212785 10.1111/j.1742-4658.2006.05558.x

[19] Schoenmakers ThJM, Visser G, Flik G, Theuvenet APR (1992) Chelator: an improved method for computing metal ion concentrations in physiological solutions. Biotech 12: 870–879. 1642895

[20] Sillen LG, Martell AE (1964) Stability constants of metal-ion complexes, The Chemical Society, Burlington House, London.

[21] Hickey RF, Vanderwielen J, Switzenbaum MS (1989) The effect of heavy metals on methane production and hydrogen and carbon monoxide levels during batch anaerobic sludge digestion. Wat Res 23: 207–218.

[22] Lane TW, Morel MM (2000) A biological function for cadmium in marine diatoms. Proc Natl Acad Sci USA 97: 4627–4631. 10781068 10.1073/pnas.090091397 PMC18283

[23] Li L, Li Q, Rohlin L, Kim U, Salmon K, et al (2007) Quantitative proteomic and microarray analysis of the archaeon *Methanosarcina acetivorans* grown with acetate versus methanol. J Proteom Res 6: 759–771. 10.1021/pr060383l PMC2577390 17269732

[24] Rohlin L, Gunsalus RP (2010) Carbon-dependent control of electron transfer and central carbon pathway genes for methane biosynthesis in the Archaean, *Methanosarcina acetivorans* strain C2A. BMC Microbiol 10: 62. 20178638 10.1186/1471-2180-10-62 PMC2838876

[25] Aceti DJ, Ferry JG (1988) Purification and characterization of acetate kinase from acetate-grown *Methanosarcina thermophila*. Evidence for regulation of synthesis. J Biol Chem 263: 15444–15448. 2844814

[26] Ingram-Smith C, Gorrell A, Lawrence SH, Iyer P, Smith K, et al (2005) Characterization of the acetate binding pocket in the *Methanosarcina thermophila* acetate kinase. J Bacteriol 187: 2386–2394. 15774882 10.1128/JB.187.7.2386-2394.2005 PMC1065240

[27] Lundie LL Jr, Ferry JG (1989) Activation of acetate by *Methanosarcina thermophila*. Purification and characterization of phosphotransacetylase. J Biol Chem 264: 18392–18396. 2808380

[28] Alber BE, Ferry JG (1994) A carbonic anhydrase from *Methanosarcina thermophila* . Proc Natl Acad Sci USA 91: 6909–6913. 8041719 10.1073/pnas.91.15.6909 PMC44307

[29] Innocenti A, Zimmerman S, Ferry JG, Scozzafava A, Supuran CT (2004) Carbonic anhydrase inhibitors. Inhibition of the zinc and cobalt gamma-class enzyme from the archaeon *Methanosarcina thermophila* with anions. Bioorg Med Chem Lett 21: 3327–3331. 10.1016/j.bmcl.2004.03.101 15149700

[30] Macauley SR, Zimmerman S, Apolinario EE, Evilia C, Hou YM, et al (2009) The archetype gamma-class carbonic anhydrase (Cam) contains iron when synthesized in vivo. Biochem 48: 817–819. 19187031 10.1021/bi802246s

[31] Tripp BC, Bell CB 3rd, Cruz F, Krebs C, Ferry JG (2004) A role for iron in an ancient carbonic anhydrase. J Biol Chem 279: 6683–6687. 14662760 10.1074/jbc.M311648200

[32] Pepi M, Baldi F (1992) Modulation of chromium (VI) toxicity by organic and inorganic sulfur species in yeasts from industrial wastes. Biomet 5: 179–185. 10.1007/BF01061326 1421967

[33] Fermoso FG, Bartacek J, Manzano R, van Leeuwen HP, Lens PNL (2010) Dosing of anaerobic granular sludge bioreactors with cobalt: Impact of cobalt retention on methanogenic activity. Biores Technol 101: 9429–9437. 10.1016/j.biortech.2010.07.053 20709540

[34] Silver S, Misra TK, Laddaga RA (1989) DNA sequence analysis of bacterial toxic heavy metal resistances. Biol Trace Elem Res 21: 145–163. 2484581 10.1007/BF02917247

[35] Karri S, Sierra-Alvarez R, Field JA (2006) Toxicity of copper to acetoclastic and hydrogenotrophic activities of methanogens and sulfate reducers in anaerobic sludge. Chemosph 62: 121–127. 10.1016/j.chemosphere.2005.04.016 15936054

[36] Kim BK, Conway de Macario E, Nolling J, Daniels L (1996) Isolation and characterization of a copper-resistant methanogen from a copper-mining soil sample. Appl Environ Microbiol 62: 2629–2635. 8779599 10.1128/aem.62.7.2629-2635.1996 PMC168042

