# Supplementary figures and images for: Activation of Methanogenesis by Cadmium in the Marine Archaeon Methanosarcina acetivorans
Source: PLoS One. 2012 Nov 12;7(11):e48779. doi: 10.1371/journal.pone.0048779 (PMC3495967; doi:10.1371/journal.pone.0048779)

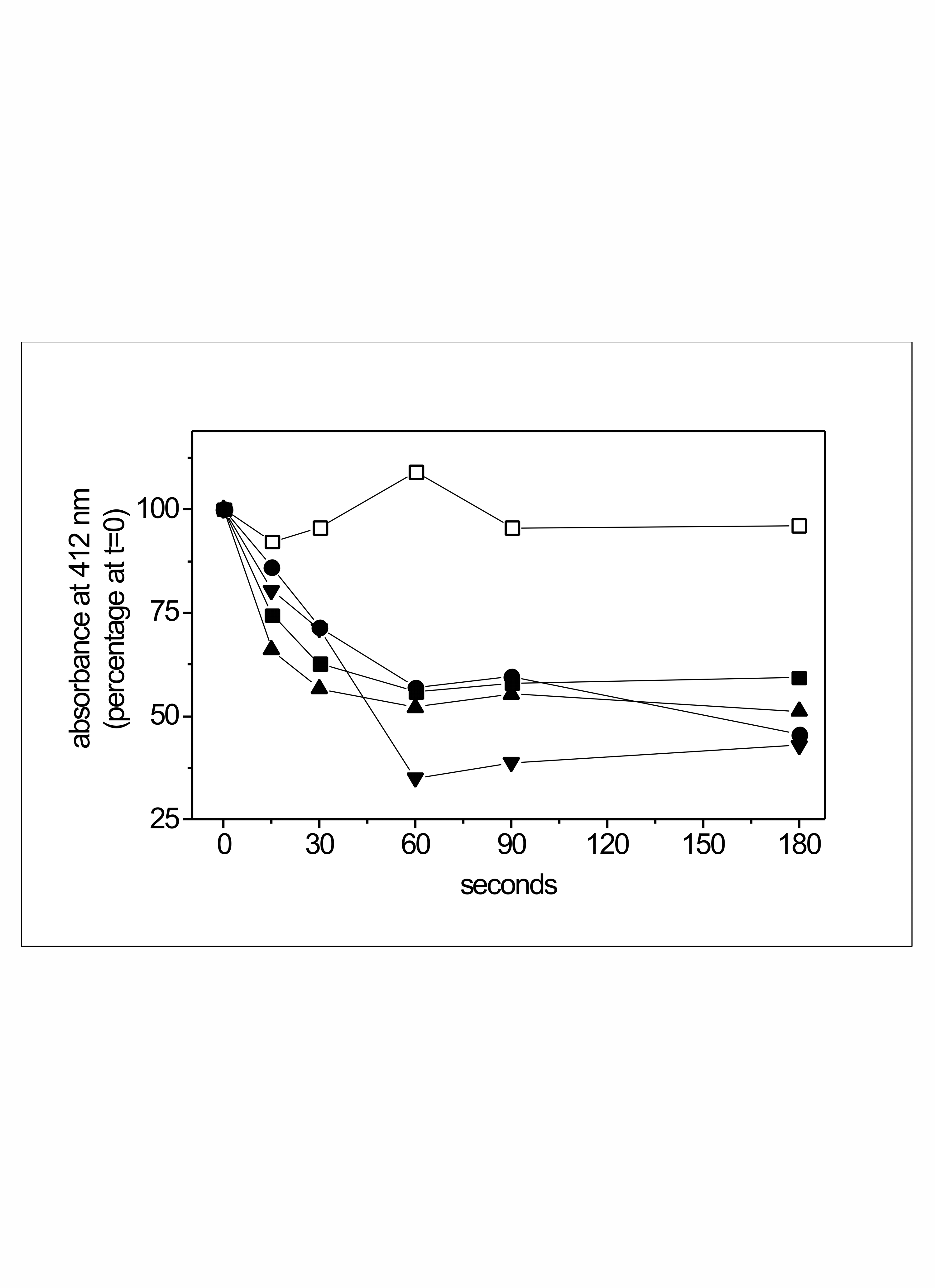

Supplement: Figure S1 — Activity of phosphotransacetylase from M. acetivorans . An aliquot of the cytosolic fraction was incubated with the substrates acetyl-Pi and CoA in the absence ((▪) or presence of 0.1 (•), 1 (▴) or 10 (▾) µM total CdCl2. In the absence of protein the CoA concentration remained constant (□). (TIF) [file pone.0048779.s001.tif]

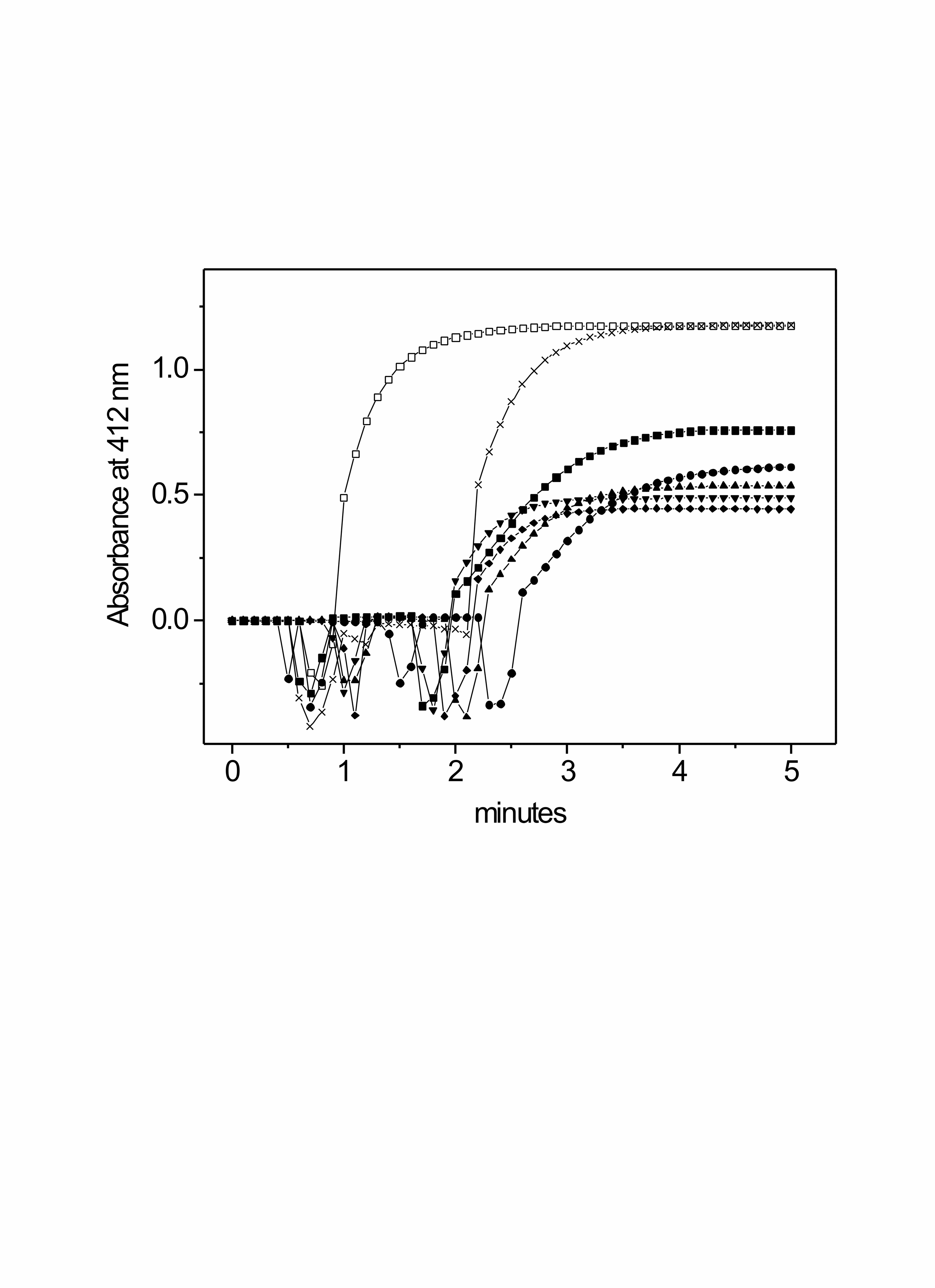

Supplement: Figure S2 — CODH/AcCoAs complex activity from M. acetivorans ; representative traces of the activity by adding cytosolic fraction (containing the enzyme complex, ferredoxin and THMPT) and 80 µM AcCoA. Representative trace with: 125 (□), 62 (x) and 25 (▪) µg of cytosolic fraction without cadmium in the absence or presence of 0.01 (•), 0.1 (▴), 1 (▾) and 10 µM total CdCl2 (⧫). (TIF) [file pone.0048779.s002.tif]

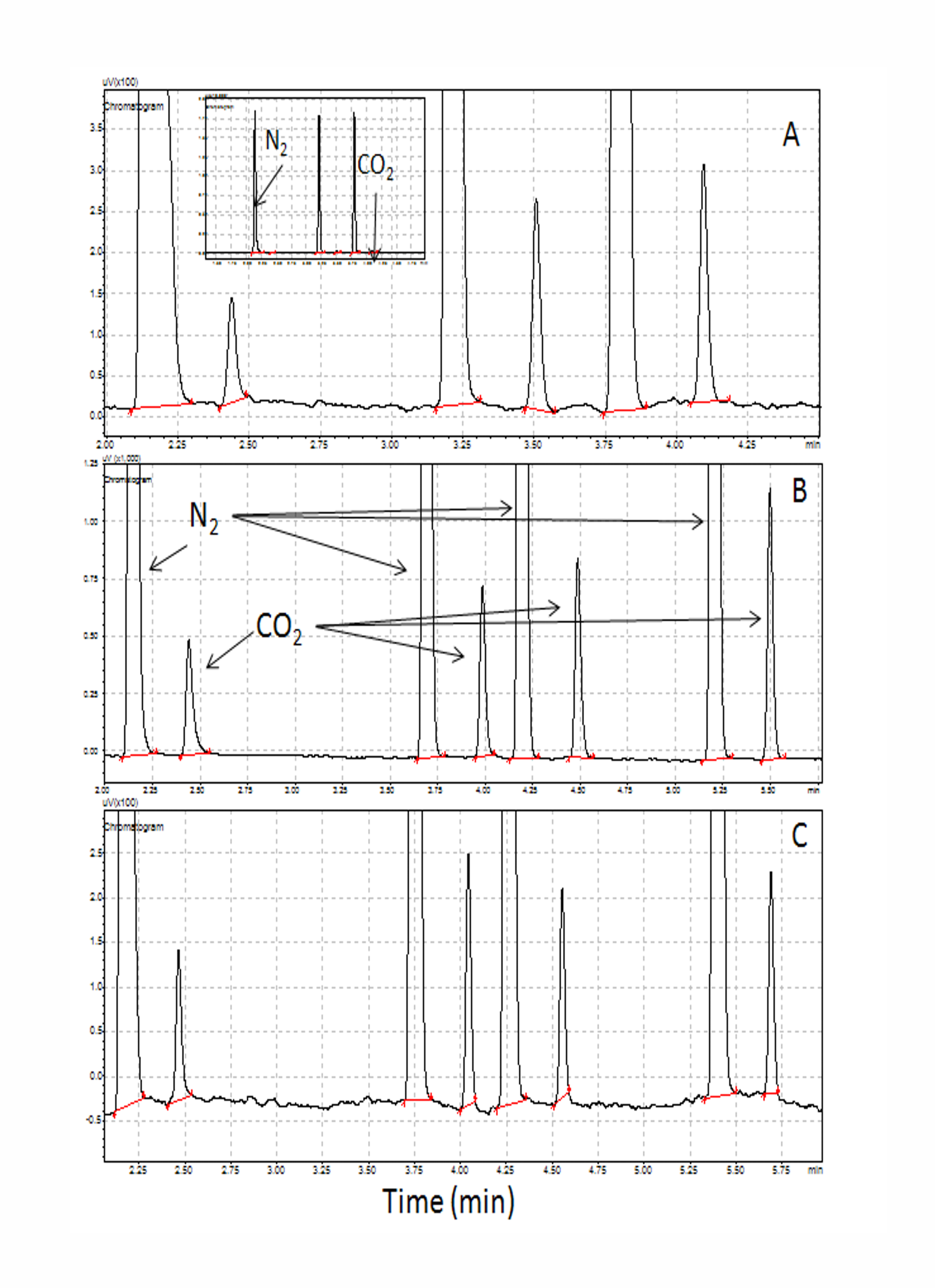

Supplement: Figure S3 — Formation of CO2 by CA from M. acetivorans in the absence (chromatogram A), or presence of 1 µM CdCl2. In chromatogram C, cytosolic fraction was previously heated. The different peaks shown represent consecutive sample injections at different times. It should be noted that y-scale is higher for chromatogram B. Under these conditions, CA activity was linear for at least 1 min (Fig. S4). Reverse reaction in turn, produced less reliable values by this method (not shown). (TIF) [file pone.0048779.s003.tif]

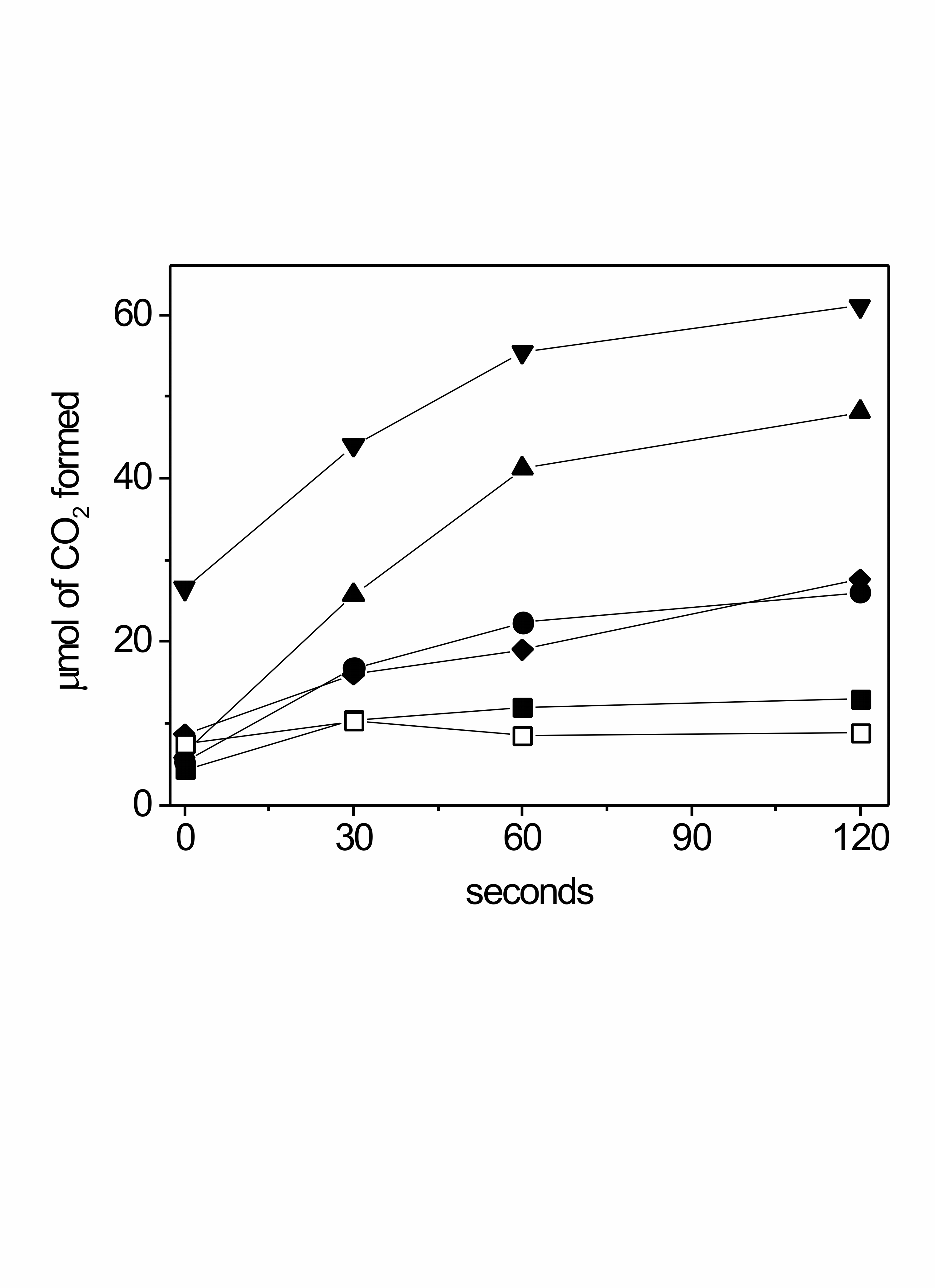

Supplement: Figure S4 — Activity of CA in the cytosolicfraction of M. acetivorans in the absence (▪) or presence of 0.01 (•), 0.1 (▴), 1 (▾) or 10 µM total CdCl2 (⧫). A representative data with heated cytosolic fraction in presence of 0.1 µM CdCl2 is also shown (□). (TIF) [file pone.0048779.s004.tif]

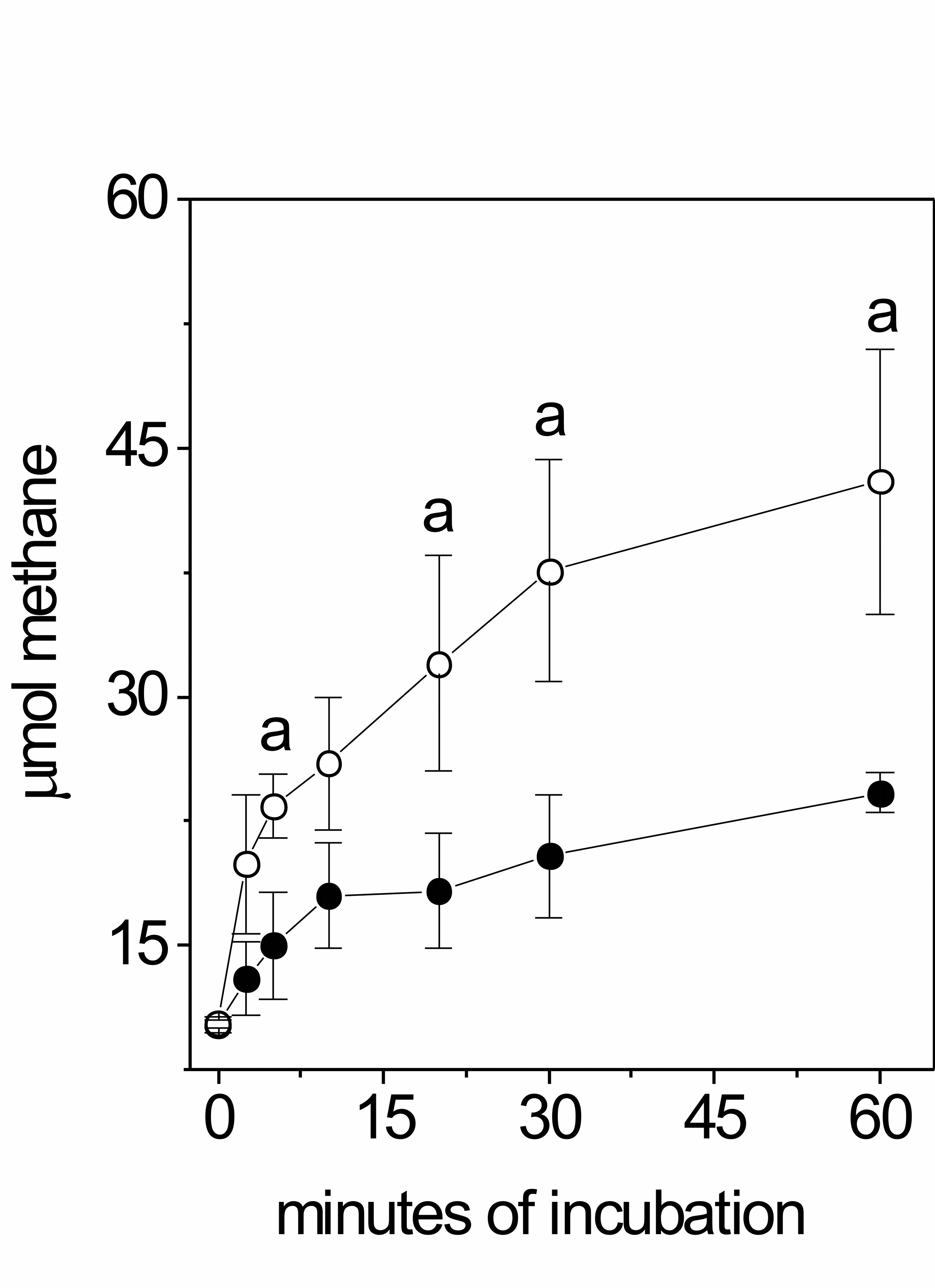

Supplement: Figure S5 — Activation of methanogenesis by cadmium. Cultures on acetate were purged by passing N2 for 5 min. Then, samples of the head space were withdrawn from the cultures at 0 and 5, 10, 20, 30 and 60 min of incubation with 0 (filled symbols) or 10 µM CdCl2 (open symbols) for GC analysis. These experiments were started with the addition of 20 mM acetate. Values are the mean ± SD of 3 independent cell preparations. P<0.05 using the Student's t-test for a vs control (without cadmium). (TIF) [file pone.0048779.s005.tif]

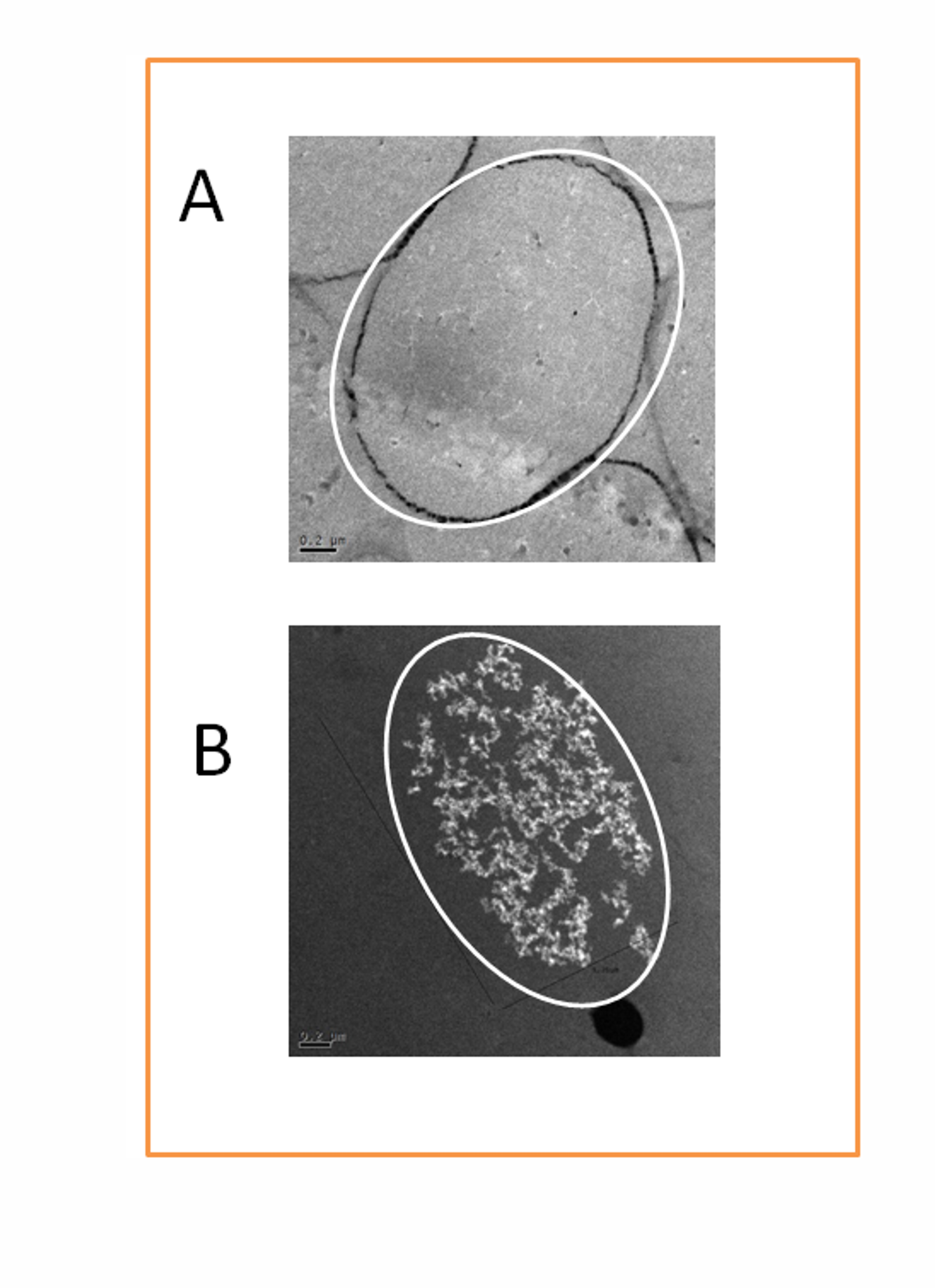

Supplement: Figure S6 — Intracellular cadmium clusters in M. acetivorans . HAADF-STEM projection images of methanol-grown cells cultured in methanol in the absence (A) or in the presence of 100 µM CdCl2 for 5 days (B). The image in B revealed cadmium grains along the cell (white spots). (TIF) [file pone.0048779.s006.tif]
