# Supplementary material for: Activation of Methanogenesis by Cadmium in the Marine Archaeon Methanosarcina acetivorans
Source: PLoS One. 2012 Nov 12;7(11):e48779. doi: 10.1371/journal.pone.0048779 (PMC3495967; doi:10.1371/journal.pone.0048779)
Supplement: Text S1 — Methods and Results. (DOCX) [file pone.0048779.s007.docx]

**Supporting information Text S1**

- **Methods S1.** Spectrophotometric determination of phosphotransacetylase activity

Phosphotransacetylase was determined measuring the remaining CoA as described in section 2.5. Figure S1 shows representative traces of cytosolic fraction with and without cadmium. Activity was linear for at least 30 s. CoA content in absence of enzyme remained constant.

- **Methods S2.** Spectrophotometric determination of CODH/AcCoAsynthease activity

The reaction catalyzed by this enzyme complex involves three substrates, AcCoA, the electron acceptor oxidized ferredoxin and the methyl carrier molecule tetrahydromethanopterin (THMPT). In our experimental conditions we assumed that the isolated cytosolic fraction contains a non-limiting concentration of ferredoxin, because ferredoxin but not F420 is present in acetate-grown cells [1]. In the same sense, the content of THMPT is high as CoM is found in *M. barkeri* [2], suggesting that the addition of AcCoA may suffice to reliably determine the complex activity. Indeed, Fig. S2 shows representative traces of CODH/AcCoAs complex activity. Linearity could be determined accurately for up to 30 s and was dependent on the amount of protein contained in the cytosolic fraction added.

- **Methods S3.** Determination of carbonic anhydrase by gas chromatography

Five μL of the head space were injected into the GC to determine the CO_2_ formed by CA activity (see section 2.5). The experimental conditions of the apparatus were: inlet temperature of 150°C, column temperature 75°C and TCD temperature of 200°C; split of 16. The pressure was 75 kPa and column flow of 1.61 mL/min at a linear velocity of 31.5 cm/sec using helium at a makeup flow of 5 mL/min. The HP-PLOT/U column under these conditions is able to accurately separate N_2_ (retention time = 2.12 min), CO_2_ (retention time= 2.4 min) and water. Because water retention time is around 7 min, up to 4 consecutive injections may be carried out in the same run. Hence, the times to determine the activity of CA were 0, 30, 60 and 120 s. As shown in Fig. S3, chromatogram A, a clear increasing CO_2_ content was attained with time. Inset shows the total set of N_2_ peaks where CO_2_ is less visible; hence, a zoom was made for presentation. The area of CO_2_ peaks increased when 1 μM CdCl_2_ was present in the mix reaction as shown in chromatogram B. When the cytosolic fraction was heated for 10 min, no increase in the CO_2_ content was observed (chromatogram C), indicating that indeed the protein CA was involved in the formation of CO_2_. The area of the CO_2_ peaks was transformed to mole of CO_2_ by using a standard curve made with 99.8 % (v/v) CO2.

**Results S1.** Activation of methanogenesis by cadmium.Cultures on acetate or methanol were purged and samples of 5 µL from the head space were withdrawn for GC analysis (Fig. S5). The split was adjusted to 25; hence, values obtained were higher than the detection limits and reliable.

**Results S2.** Identification of cadmium clusters in *M. acetivorans*

The white and dense grains are showing the cadmium accumulated into cells (Fig. S6). Interestingly, sulfur was also detected in these grains suggesting that molecules with thiol groups such as cysteine, sulfide and coenzyme M may be forming complexes with cadmium for its sequestration and inactivation.

**Supplementary References**

[1] Wang, M., Tomb, J. F., and Ferry, J. G. (2011) [Electron transport in acetate-grown *Methanosarcinaacetivorans*.](http://www.ncbi.nlm.nih.gov/pubmed/21781343)BMC Microbiol.**11**: 165.

[2] Feist, A. M., Scholten, J. C., Palsson, B. Ø., Brockman, F. J., and Ideker, T. (2006) [Modeling methanogenesis with a genome-scale metabolic reconstruction of *Methanosarcinabarkeri*.](http://www.ncbi.nlm.nih.gov/pubmed/16738551)MolSystBiol**2**: 2006.0004.
